# Elective caesarean section versus vaginal delivery for preventing mother to child transmission of hepatitis B virus – a systematic review

**DOI:** 10.1186/1743-422X-5-100

**Published:** 2008-08-28

**Authors:** Jin Yang, Xue-mei Zeng, Ya-lin Men, Lian-san Zhao

**Affiliations:** 1 Center of Infectious Diseases, National Key Laboratory of Biotherapy for Human Diseases, West China Hospital of Sichuan University, Chengdu 610041, Sichuan Province, PR China

## Abstract

**Background:**

Caesarean section before labor or before ruptured membranes ("elective caesarean section", or ECS) has been introduced as an intervention for preventing mother-to-child transmission (MTCT) of hepatitis B virus (HBV). Currently, no evidence that ECS versus vaginal delivery reduces the rate of MTCT of HBV has been generally provided. The aim of this review is to assess, from randomized control trails (RCTs), the efficacy and safety of ECS versus vaginal delivery in preventing mother-to-child HBV transmission.

**Results:**

We searched Cochrane Pregnancy and Childbirth Group's Trials Register (January, 2008), the Cochrane Central Register of Controlled Trials (the Cochrane Library 2008, issue 1), PubMed (1950 to 2008), EMBASE (1974 to 2008), Chinese Biomedical Literature Database (CBM) (1975 to 2008), China National Knowledge Infrastructure (CNKI) (1979 to 2008), VIP database (1989 to 2008), as well as reference lists of relevant studies. Finally, four randomized trails involving 789 people were included. Based on meta-analysis, There was strong evidence that ECS versus vaginal delivery could effectively reduce the rate of MTCT of HBV (ECS: 10.5%; vaginal delivery: 28.0%). The difference between the two groups (ECS *versus *vaginal delivery) had statistical significance (RR 0.41, 95% CI 0.28 to 0.60, P < 0.000001). No data regarding maternal morbidity or infant morbidity according to mode of delivery were available.

**Conclusion:**

ECS appears to be effective in preventing MTCT of HBV and no postpartum morbidity (PPM) was reported. However, the conclusions of this review must be considered with great caution due to high risk of bias in each included study (graded C).

## Background

### Description of the condition

Hepatitis B is a major global problem. More than two billion people alive today have been infected worldwide [1] and approximately 350 million people are chronically infected with hepatitis B virus (HBV) [2,3]. Chronic hepatitis B (CHB) is associated with serious complications, including liver failure, cirrhosis, and hepatocellular carcinoma [4-6]. Each year more than one million patients with CHB worldwide die from these diseases [1].

Mother-to-child transmission(MTCT) of HBV is one of the most important causes of chronic HBV infection [7-9] and remains one serious problem despite passive immunization (hepatitis B immune globulin at birth) and active immunization (hepatitis B vaccination according to the standard 3-dose schedule). MTCT may occur prenatally, during delivery, or postpartum. Currently, a series of measures have been taken to prevent both prenatal and postpartum routes of transmission with progress being achieved to some extent. However, with regard to MTCT of HBV during delivery, disagreements still exist on the issue of whether different mode of delivery (mainly caesarean section *versus *vaginal delivery) will affect the risk of mother-to-child HBV transmission [10,11].

### Description of the intervention

Of the cases of MTCT of HBV, a large population occur during the intrapartum period. Underlying mechanisms may include transfusion of the mother's blood to the fetus during labor contractions, infection after the rupture of membranes, and direct contact of the fetus with infected secretions or blood from the maternal genital tract [12-16]. As elective caesarean section (ECS) is performed before the onset of labor or the rupture of membranes, it could effectively avoid the disbenefits described above. Therefore, ECS might reduce the risk of MTCT of HBV (compared with vaginal delivery or cesarean section after onset of labor or after rupture of membranes).

It is well known that, in the absence of HBV infection, ECS is related to increased risks of maternal and infant morbidity [17-19]. In a population of HBV-infected women, the procedure would be expected to be associated with the same or greater deleterious effects on both mother and infant. For instance, surgical delivery would be expected to increase the risk of fever, endometritis, and hemorrhage and severe anemia among women. Commonly, Infants born by ECS at term are at increased risk for developing respiratory disorders compared with those born by vaginal delivery. However, although the risk of neonatal respiratory morbidity is higher, the number of affected infants is small [20]. In addition to respiratory morbidity (respiratory distress syndrome, transient tachypnea of the newborn), an increased risk of lacerations of newborn skin is also of concern with surgical delivery.

### Why it is important to do this overview

Given the uncertainty of findings from current studies, what is more, HBV-infected pregnant women must be provided with available information with which to make informed decisions regarding ECS and other options to prevent transmission of infection to their children, we aim to determine if there is any evidence from randomized controlled trials (RCTs) that offering ECS to mothers who are infected with HBV affects the risk of MTCT of HBV.

## Results

### Description of studies

#### Studies identified

A total of 942 studies were identified by the searches. No unpublished studies or other information was obtained from contact with WHO and individual researchers. By scanning titles and abstracts of the 942 studies, 927 studies, including overlapped studies, reviews, case reports and meta analyses, were excluded. After referring to full texts, 11 studies were excluded upon further scrutiny due to the following reasons: 7 studies made HBV-infected women with positive hepatitis B surface antigen (HBsAg) and/or hepatitis B e antigen (HBeAg) as their participant criteria; 3 studies had other interventions potentially impacting the outcome; 1 study did not provide data to meet the outcome criteria. Finally, we included 4 studies, involving 789 people, which were all performed in china. Among them, 1 were published in English [21] (Lee 1988), 3 in Chinese (Ji 2002, Wang 2004, Liu 2008, available only by searching the database of CNKI). Apart from Chinese and English, we did not search citations in other languages.

#### Designs of included studies

All the included studies were of a parallel design, single centre and had a control group.

#### Participants of included studies

Numbers of participants of the individual studies ranged from 97 to 244 with a total of 789 participants included in this review. All of them were HBV-infected pregnant women with HBV DNA-positive in sera of blood. The baseline characteristics (including the age, race, gravidity, parity, pregnant week, disease duration, and severity of disease, etc) were similar in the two groups (P > 0.05).

#### Interventions of included studies

ECS was made as the intervention group, and vaginal delivery as the control group in each of the four studies.

#### Outcomes of included studies

None of the four studies reported the maternal morbidity or infant morbidity associated with ECS. The common outcome reported was the positive rate of HBV DNA in neonates under different mode of delivery (ECS *versus *vaginal delivery).

### Methodological quality

#### Randomization

All the four studies mentioned "random", "randomize" or "randomized", but did not give a clear description of the randomization procedure.

#### Allocation concealment

No allocation concealment was used in each of the four studies

#### Blinding

No blind was used in each of the four studies

#### Description of withdrawals, dropouts, losses of follow up and intention-to-treat analysis

Neither of the included studies mentioned withdrawals, dropouts, losses of follow up or performed any intention-to-treat analysis.

According to the quality criteria listed above, we considered each included study was at high risk of bias and graded as category C.

### Effects of interventions

#### Assessment of the efficacy of ECS versus vaginal delivery for preventing MTCT of HBV

**Figure 1:**
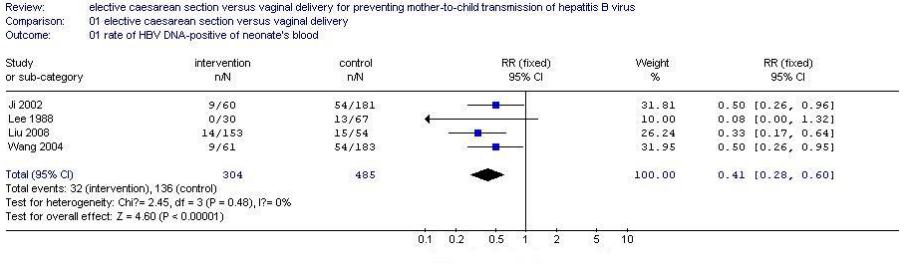
Analysis of the efficacy of ECS versus vaginal delivery for preventing MTCT of HBV.

Four studies demonstrated the efficacy of ECS compared to vaginal delivery for the prevention of MTCT of HBV. According to chi-squared statistic and I square (I^2^), the results of the four studies showed no statistical heterogeneity (p = 0.48.I^2 ^= 0%). So we used fixed effect model for meta-analysis. After synthesizing the results, we found out that the rate of MTCT of HBV according to mode of delivery differed significantly (ECS: 10.5%; vaginal delivery: 28.0%). The difference between the two groups (ECS *versus *vaginal delivery) had statistical significance (RR 0.41, 95% CI 0.28 to 0.60, P < 0.000001) (Figure 1). Therefore, in comparison to vaginal delivery, ECS is more efficacious for the prevention of MTCT of HBV.

#### Subgroup analyses

**Figure 2:**
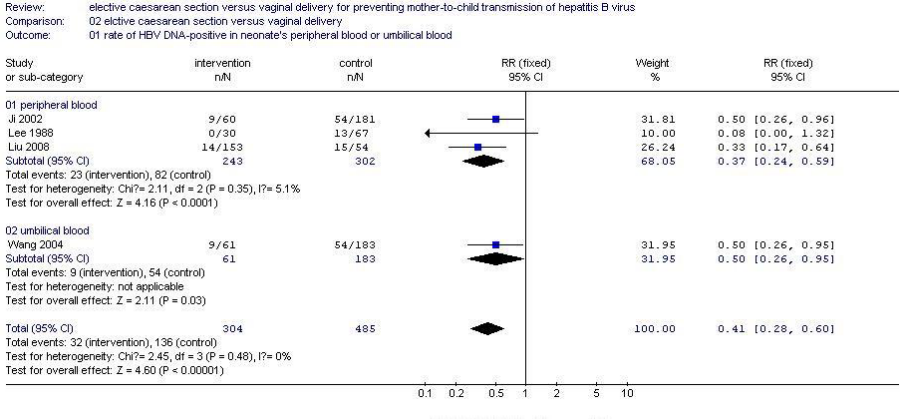
Subgroup analysis of the efficacy of ECS versus vaginal delivery for preventing MTCT of HBV.

Of the four included studies, one trail performed the detection of HBV DNA in neonate's umbilical blood, while other three trails in neonate's peripheral blood. So subgroup analysis was carried out under the two circumstances. The trend towards elevated rate of MTCT of HBV in the ECS group compared to vaginal delivery group with HBV DNA detected in neonate's umbilical blood (RR 0.50, 95% CI 0.26 to 0.95) was similar to studies with HBV DNA detected in neonate's peripheral blood (RR 0.37, 95% CI 0.24 to 0.59) (Figure 2).

### Sensitivity analyses

We did not carry out any of the planned sensitivity analyses as no unpublished studies were found and all included studies were at high risk of bias (graded C).

### Assessment of publication bias

There was an insufficient number of trials for us to assess publication bias.

### Adverse events

No postpartum morbidity (PPM) associated with ECS was reported.

## Discussion

### Analysis of the effect of ECS for preventing MTCT of HBV

Four clinical trails were identified which evaluated the efficacy of ECS for the prevention of MTCT of HBV. They indicate ECS could significantly reduce the risk of MTCT. With regard to postpartum morbidity (PPM), none of these studies had reported the maternal morbidity or infant morbidity associated with ECS. Therefore, based on the included studies, the benefit of ECS outweighs the risk of PPM among HBV-infected women. However, it is important to point out that the risk/benefit ratio should depend on the underlying rate of MTCT. With very low rates of MTCT, the risks associated with ECS among HBV-infected women may outweigh the benefits.

Currently, HBV DNA-positive is mostly considered the direct index reflecting the infectivity of HBV. So we only included HBV-infected women with HBV DNA-positive, and also made neonates with HBV DNA-positive as the outcome criteria. However, the detailed HBV DNA levels in included HBV-infected women were not described, except one study (Liu 2008), which included patients with HBV DNA > 10^5 ^copy/ml. So we can't get adequate information for the magnitude of infectivity, which may influence the effect of ECS for the prevention of MTCT of HBV in this review. Subgroup analysis demonstrates that, HBV DNA-positive either in neonate's umbilical blood or in neonate's peripheral blood indicates the existence of HBV infection, but false positive must be excluded as a result of nonstandard collection of blood. According to principles of medical ethics, we suggest neonate's umbilical blood should be chosen for detection of HBV DNA, which can largely relieve pains of newborns. But it must be performed strictly to avoid pollution by maternal blood.

### Limitations of this systematic review

The conclusions of this review must be considered with great caution.

All the retrieved studies did not give adequate descriptions of the methodology used, which may have misled us if we had not clarified the details, for example, inclusion of non-RCTs and classifying the trials into category B rather than C. It was an exhausting but necessary process to interview every primary trial author before deciding whether to include these trials, when the methodological details were not reported. Contacting authors by telephone was more effective than writing to them because of a higher response rate and left no time for the trial authors to make up artificial details. However, even after confirmation of true randomization, we found that the methodological quality of these studies remained poor.

Allocation concealment is important in preventing selection bias. Each of the studies related to our question did not use any approach to conceal the allocation process, which could lead to a high risk of selection bias.

No blinding was conducted with either the participants or the investigators, which led to a high risk of performance bias. None of the studies mentioned blinding to the outcome assessors, which promotes suspicion of detection bias. Publication bias may exist as no primary articles reporting negative results were found. No information of numbers of withdrawals, dropouts, losses of follow up may have led to high attrition bias in one study.

In addition, included studies of ECS among HBV-infected women have been conducted exclusively in china. In other countries, the risks and benefits associated with ECS have been largely unexplored.

## Conclusion

### Implications for practice

Although studies of ECS showed a strong evidence of a reduction in the risk of MTCT of HBV, methodological concerns including lack of information on randomization procedure, lack of allocation concealment, and lack of blinding, make the role of ECS for preventing MTCT of HBV uncertain.

### Implications for research

More high quality controlled trials are required for assessing the effects of ECS in comparison to vaginal delivery for preventing MTCT of HBV. We suggest that well-designed RCTs with adequate power to provide a definitive answer, need be conducted. The randomization procedure should be clearly described. Allocation concealment should be emphasized and the approaches should be reported. Blinding should be conducted, though this may be difficult. Additionally, more attention should be paid to PPM associated with ECS.

## Methods

### Criteria for considering studies for this review

#### Types of studies

We included RCTs only.

#### Types of participants

HBV-infected Pregnant women with HBV DNA-positive (HBV DNA > 10^3^copies/ml) in sera of blood and their babies.

#### Types of intervention

ECS versus vaginal delivery.

#### Types of outcome measures

Primary outcomes (HBV transmission-related)

HBV-infection in neonates: HBV DNA-positive in umbilical blood or peripheral blood after birth.

Secondary outcomes (morbidities related to the actual method of delivery)

(1)Maternal morbidity: types of maternal morbidity evaluated includes: febrile morbidity, endometritis, hemorrhage or severe anemia, pneumonia, and urinary tract infections.

(2)Infant morbidity: types of infant morbidity evaluated includes: respiratory morbidity (respiratory distress syndrome and transient tachypnea of the newborn) and skin lacerations.

### Search methods for identification of studies

#### Electronic searches

We searched the electronic databases as follows: Cochrane Pregnancy and Childbirth Group's Trials Register (January, 2008), the Cochrane Central Register of Controlled Trials (the Cochrane Library 2008, issue 1), PubMed (1950 to 2008), EMBASE (1974 to 2008), Chinese Biomedical Literature Database (CBM) (1975 to 2008), China National Knowledge Infrastructure (CNKI) (1979 to 2008), VIP database (1989 to 2008). We also searched additional trials by scanning the reference lists of relevant trials identified. The search strategy was iterative as follows:

1 HBV

2 HBV INFECT*

3 HBV INFECTIONS

4 HBV INFECTED

5 #1 OR #2 OR #3 OR #4

6 DELIVERY, OBSTETRIC

7 DELIVERY AND PREGNANCY

8 CAESAREAN SECTION

9 "MODE OF DELIVERY" AND PREGNANCY

10 #6 OR #7 OR #8 OR #9

11 INFANT MORTALITY

12 INFANT MORBIDITY

13 NEONATAL MORTALITY

14 NEONATAL MORBIDITY

15 MATERNAL MORTALITY

16 MATERNAL MORBIDITY

17 POSTPARTUM MORTALITY

18 POSTPARTUM MORBIDITY

19 #11 OR #12 OR #13 OR #14 OR #15 OR #16 OR #17 OR#18

20 #5 AND #10 AND #19

21 (ANIMAL OF ANIMALS) NOT HUMAN

22 #20 NOT #21

#### Other search strategies

Organizations (including the World Health Organization), individual researchers working in the field were contacted in order to obtain possible additional references, unpublished trials, or ongoing trials, confidential reports and raw data of published trials.

### Selection of studies

The titles, abstracts and keywords of every record retrieved were scanned to determine which were possibly relevant to the review. Any record that appeared likely to meet the inclusion criteria was obtained in full text. If there was any doubt regarding eligibility from the information given in the title and abstract, the full article was retrieved for clarification. Differences in opinion between reviewers were resolved by discussion.

### Data extraction

Two review authors (ZX, MY) independently extracted data concerning details of the study population, interventions and outcomes using a standard data extraction form, specifically designed for this review. We resolved differences in data extraction by consensus, and with reference to the original article. If necessary, we sought information from the authors of the primary studies. For dichotomous outcomes, number of events and total number in each group were extracted. For continuous outcomes, mean, standard deviation and sample size of each group were extracted.

### Assessment of risk of bias in included trials

The risk of bias was assessed based largely on the quality criteria specified by the Cochrane Handbook for Systematic Reviews of Interventions 5.0.0 [22]. In particular, the following factors were studied:

• Selection bias: a) was the randomization procedure adequate? b) was the allocation concealment adequate?

• Performance bias: were the patients and people performing the intervention blind to the intervention?

• Attrition bias: a) were withdrawals, dropouts and losses of follow-up completely described? b) was analysis performed by intention-to-treat?

• Detection bias: were outcome assessors blind to the intervention?

Based on these criteria, studies were broadly divided into the following three categories. This classification was used as the basis of a sensitivity analysis. Additionally, we intended to explore the influence of individual quality criteria in a sensitivity analysis.

• A: all quality criteria met – low risk of bias.

• B: one or more of the quality criteria only partly met-moderate risk of bias.

• C: one or more criteria not met – high risk of bias.

Each trial was assessed by two reviewers independently (ZX, MY). Disagreements were resolved, where necessary, by recourse to a third reviewer (YJ). In cases of disagreement, the rest of the group were consulted and a judgment was made based on consensus.

### Data Analysis

Statistical analysis was carried out by using Review Manager (version 4.2). Dichotomous data were presented as relative risk (RR) and continuous outcomes as weighted mean difference (WMD), both with 95% confidence intervals (CI). The overall effect was tested by using Z score with significance being set at P < 0.05. Heterogeneity was tested by using the chi-squared statistic and I square (I^2^) with significance being set at P < 0.1. Possible sources of heterogeneity were to be assessed by sensitivity and subgroup analyses. A fixed-effect model was to be used when the studies in the subgroup were sufficiently similar (P > 0.10, I^2 ^< 50). A random effects model was to be used in the summary analysis when there was heterogeneity between the subgroups. Publication bias was to be tested by using the funnel plot or other corrective analytical method, depending on the number of clinical trials included in the systematic review.

## Competing interests

The authors declare that they have no competing interests.

## Authors' contributions

JY conceived the study and made substantial contributions to its design, acquisition, analysis and interpretation of data. XZ and YM participants in the design, acquisition, analysis and interpretation of data. LZ participated in the design and revised the manuscript critically for important intellectual content. All authors gave final approval of the version to be submitted and any revised version.

## References

[B1] Hepatitis B Fact Sheet WHO/204 2000. http://www.who.int/mediacentre/factsheets/fs204/en.

[B2] Purcell RH (1993). The discovery of the hepatitis viruses. Gastroenterology.

[B3] Lee WM (1997). Hepatitis B virus infection. N Engl J Med.

[B4] Liaw YF, Tai DI, Chu CM, Chen TJ (1988). The development of cirrhosis in patients with chronic type B hepatitis: a prospective study. Hepatology.

[B5] Beasley RP (1988). Hepatitis B virus: the major etiology of hepatocellular carcinoma. Cancer.

[B6] Maddrey WC (2000). Hepatitis B: an important public health issue. J Med virol.

[B7] Schweitzer IL (1975). Vertical transmission of hepatitis B surface antigen. Am J Med Sci.

[B8] Mushahwar IK, Drenstag JL, Pollesky HF, McGrath LC, Decker RH, Overby LR (1981). Interpretation of various serological profiles of hepatitis B virus infection. Am J Clin Path.

[B9] Ghendon Y (1990). WHO strategy for the global elimination of new cases of hepatitis B. Vaccine.

[B10] Liu HH, Kao JH, Hsu HY, Mizokami M, Hirano K, Chen DS (1996). Least microtransfusion from mother to fetus in elective cesarean delivery. Obstetrics & Gynecology.

[B11] Wang J, Zhu Q, Zhang X (2002). Effect of delivery mode on maternal-infant transmission of hepatitis B virus by immunoprophylaxis. Chin Med J (Engl).

[B12] Stevens CE, Beasley RP, Tsui J, Lee W-C (1975). Vertical transmission of hepatitis B antigen in Taiwan. N Engl J Med.

[B13] Lee AKY, Ip HMH, Wong VCW (1978). Mechanisms of maternal-fetal transmission of hepatitis B virus. J Infect Dis.

[B14] Woo D, Cummins M, Davies PA, Harvey DR, Hurley R, Waterson AP (1979). vertical transmission of hepatitis B surface antigen in carrier mothers in two west London hospitials. Arch Dis Child.

[B15] Wong VC, Lee AK, Ip HM (1980). Transmission of hepatitis B antigens from symptom free carrier mothers to the fetus and the infant. Br J Obstet Gynaecol.

[B16] Gilbert GL (1981). Vertical transmission of hepatitis B: Review of the literature and recommendations for management. Med J Aust.

[B17] Petitti DB (1985). Maternal mortality and morbidity in cesarean section. Clin Obstet Gynecol.

[B18] Miller JR (1988). Maternal and neonatal morbidity and mortality in cesarean section. Obstet Gynecol Clin North Am.

[B19] Morrison JJ, Rennie JM, Milton PJ (1995). Neonatal respiratory morbidity and mode of delivery at term: influence of timing of elective caesarean section. Br J Obstet Gynaecol.

[B20] Zanardo V, Simbi AK, Franzoi M, Soldá G, Salvadori A, Trevisanuto D (2004). Neonatal respiratory morbidity risk and mode of delivery at term: influence of timing of elective caesarean delivery. Acta Paediatr.

[B21] Lee SD, Lo KJ, Tsai YT, Wu JC, Wu TC, Yang ZL, Ng HT (1988). Role of caesarean section in prevention of mother-infant transmission of hepatitis B virus. Lancet.

[B22] Higgins JPT, Green S (2008). Cochrane Handbook for Systematic Reviews of Interventions Version 5.0.0 [updated February 2008]. The Cochrane Collaboration.

